# Facilitators and Barriers to the Implementation of Function and Behavior Focused Care

**DOI:** 10.1093/geroni/igaa057.2808

**Published:** 2020-12-16

**Authors:** Erin Vigne, Elizabeth Galik, Barbara Resnick

**Affiliations:** 1 University of Maryland School of Nursing, Baltimore, Maryland, United States; 2 University of Maryland School of Nursing, Ellicott City, Maryland, United States

## Abstract

Function and Behavior Focused Care (FBFC) is a two tiered intervention based on the Social Ecological Model and Social Cognitive Theory that focuses on training long term care staff to cue, model, and assist residents with dementia to engage in physical activity and perform functional tasks. FBFC was implemented by a Research Nurse and a facility based Champion for 12 months and involved a four step approach: (1) Environment and Policy Assessments; (2) Staff Education; (3) Establishing Resident Care Plans; and (4) Mentoring and Motivating of Staff and Residents. Facilitators of implementation included administrative support, an identified champion, and supportive environments and policies. Challenges and barriers included the fear of falls, fear of behavioral symptom exacerbation, competing priorities, and lack of facility specific goals. Innovative approaches to overcoming these barriers will be reviewed.

